# Respiratory variability in mechanically ventilated patients

**DOI:** 10.1186/cc9621

**Published:** 2011-03-11

**Authors:** T Desaive, L Piquilloud, K Moorhead, J Roeseler, JG Chase, E Bialais, PF Laterre, P Jolliet, T Sottiaux, D Tassaux, B Lambermont

**Affiliations:** 1 University of Liege, Belgium; 2 University Hospital, Lausanne, Switzerland; 3 Cliniques Universitaires St-Luc, Brussels, Belgium; 4 University of Canterbury, Christchurch, New Zealand; 5 La clinique Notre Dame de Grâce, Gosselies, Belgium; 6 University Hospital, Geneva, Switzerland

## Introduction

Increased respiratory pattern variability is associated with improved oxygenation. Pressure support (PS) is a widely used partial-assist mechanical ventilation (MV) mode, in which each breathing cycle is initiated by flow or pressure variation at the airway due to patient inspiratory effort. Neurally adjusted ventilatory assist (NAVA) is relatively new and uses the electrical activity of the diaphragm (Eadi) to deliver ventilatory support proportional to the patient's inspiratory demand. We hypothesize that respiratory variability should be greater with NAVA compared with PS.

## Methods

Twenty-two patients underwent 20 minutes of PS followed by 20 minutes of NAVA. Flow and Eadi curves were used to obtain tidal volume (Vt) and ∫Eadi for 300 to 400 breaths in each patient. Patient-specific cumulative distribution functions (CDF) show the percentage Vt and ∫Eadi within a clinically defined (±10%) variability band for each patient. Values are normalized to patient-specific medians for direct comparison. Variability in Vt (outcome) is thus expressed in terms of variability in ∫Eadi (demand) on the same plot.

## Results

**Figure 1:**
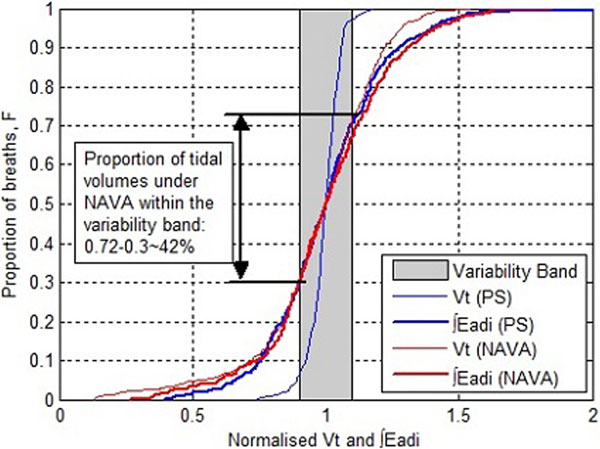


Variability in Vt relative to variability in ∫Eadi is significantly greater for NAVA than PS (*P *= 0.00012). Hence, greater variability in outcome Vt is obtained for a given demand in ∫Eadi, under NAVA, as illustrated in Figure 1 for a typical patient. A Fisher 2 × 2 contingency analysis showed that 45% of patients under NAVA had a Vt variability in equal proportion to ∫Eadi variability, versus 0% for PS (*P *< 0.05).

## Conclusions

NAVA yields greater variability in tidal volume, relative to ∫Eadi demand, and a better match between Vt and ∫Eadi. These results indicate that NAVA could achieve improved oxygenation compared with PS when sufficient underlying variability in ∫Eadi is present, due to its ability to achieve higher tidal volume variability from a given variability in ∫Eadi.

